# Allied male dolphins use synchronous displays to strengthen social bonds in a cooperative context

**DOI:** 10.1186/s40462-025-00603-z

**Published:** 2025-11-21

**Authors:** Sam Hill-Cousins, Emma Chereskin, Simon J. Allen, Richard C. Connor, Michael Krützen, Danai Papageorgiou, Stephanie L. King

**Affiliations:** 1 School of Biological Sciences, University of Bristol, Bristol, BS8 1TQ UK; 2 Evolutionary Genetics Group, Department of Evolutionary Anthropology, University of Zurich, Zurich, CH-8057 Switzerland; 3 School of Biological Sciences, Oceans Institute, University of Western Australia, Perth, WA 6009 Australia; 4 Institute of Environment & Department of Biological Sciences, Florida International University, Miami, FL 33181 USA

**Keywords:** Synchrony, Rhythm, Cooperation, Motor coordination, Social bonding, Alliance formation, Bottlenose dolphins, Collective behaviour, Drones, Behavioural tracking, Video analysis

## Abstract

From fireflies to fiddler crabs and anurans to apes, males from across the animal kingdom perform synchronous displays in the mating context. Most do so in competition for mates, but few species synchronise signal production cooperatively. For example, synchrony in humans is cooperative; where interpersonal synchrony increases affiliation, cooperative ability, and group cohesion. Interestingly, multi-level alliances of male Indo-Pacific bottlenose dolphins in Shark Bay, Western Australia, also engage in cooperative synchrony. Adult males form pairs or trios to cooperatively herd single oestrus females, during which males perform elaborate synchronous displays. These displays comprise synchronous turns, leaps, and surfacing bouts around the female, but the function remains equivocal. Here, we used drone mounted video coupled with 40 years of behavioural data to investigate what social factors influence the performance of these synchronous displays by male dolphins. We found that males perform synchronous displays for longer after engaging in affiliative contact behaviour, and that those with weaker social bonds engaged in more precise synchrony with each other. Aligning with recent research on vocal synchrony in this population, we provide evidence that male dolphins use synchrony to both maintain and strengthen their social bonds. These findings suggest that dolphins have converged with humans on the use of synchronous displays to sustain multiple social bonds in nested, complex social systems.

**Supplementary Information:**

The online version contains supplementary material available at 10.1186/s40462-025-00603-z.

## Introduction

Synchronous group displays, where individual animals precisely synchronise signals, can be found in humans, arthropods and anurans [1]. Many species of male katydids, for example, produce synchronous choruses of chirps when attracting mates [2]. Similarly, male fiddler crabs will synchronously wave their enlarged claws as females approach [3]. In these cases, synchrony is an epiphenomenon of male competition; katydids attempt to produce a leading chirp [2], and fiddler crabs compete to lead a wave [3]. In contrast to such competitive synchrony, several firefly species cooperatively and precisely synchronise their flashes, likely as a means of intensifying the signal to attract females [4–6].

Human synchrony is also highly cooperative [7–11]. Experiments manipulating whether participants walk or tap in synchrony find that motor synchrony increases feelings of affiliation and trust between synchronous partners [7, 8, 11]. This rise in rapport fosters cooperation within synchronous groups, who outcompete non synchronous groups in subsequent economic games [11]. Synchronous groups are also perceived as being more unified and capable [12–14] and the participants themselves will perceive threats as less formidable [15]. Accordingly, it becomes clear why many distinct cultural groups have incorporated synchrony into rituals and ceremonies, and why military forces worldwide march in step; in humans, synchrony is a social tool that increases group rapport, fosters cooperation and signals alliance unity [7–16].

**Fig. 1:**
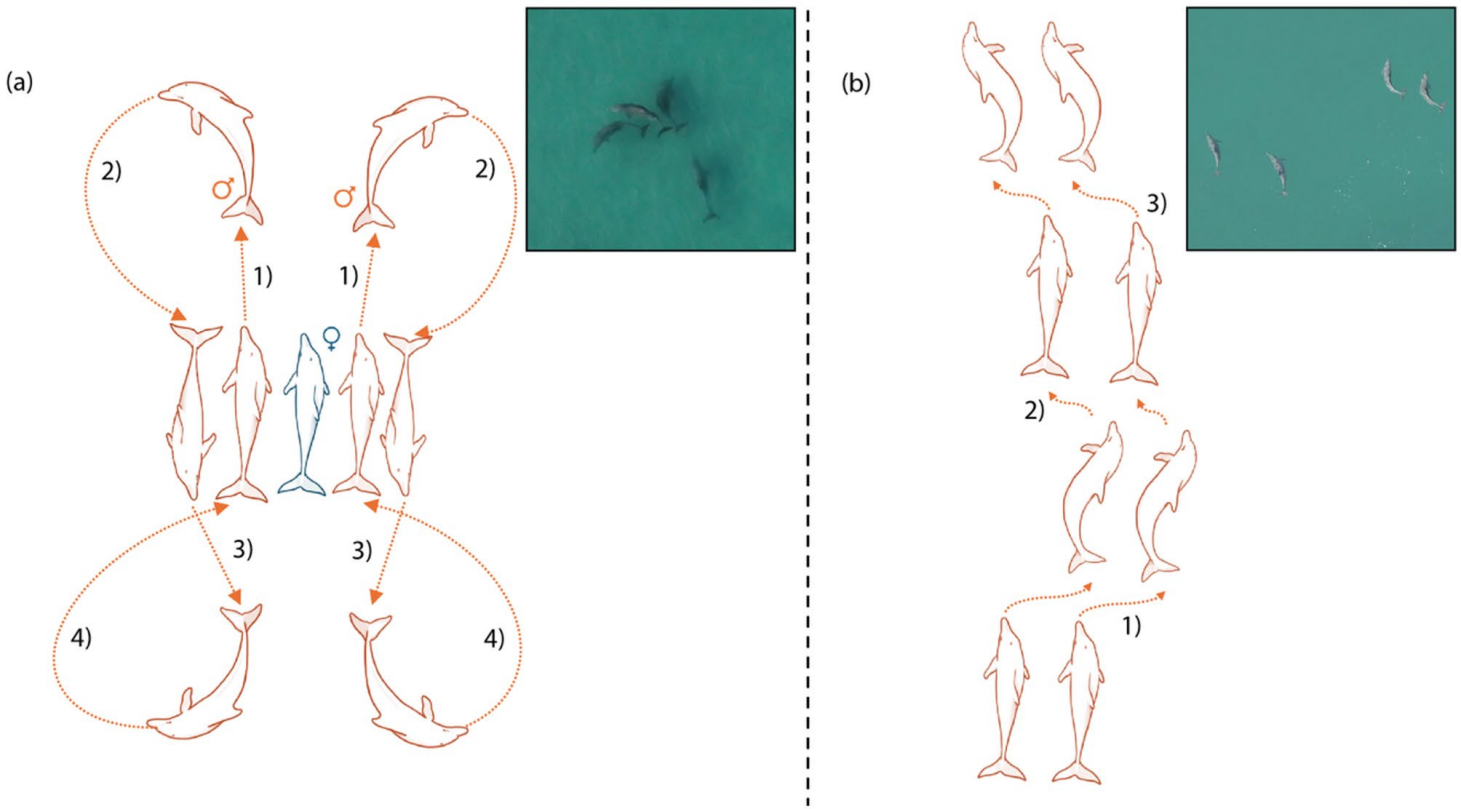
Stereotypical structure of (**a**) butterfly and (**b**) tango synchronous displays. Male dolphins coloured red and female blue. (**a**) General pattern of a butterfly display where (1) males swim out in front of the female, before (2) turning in front of her to (3) swim past her before (4) turning back into the starting position. (**b**) General pattern of a tango display, where males swimming parallel to each other will (1) synchronously turn < 90 degrees, before (2) straightening up and then (3) quickly executing another < 90-degree synchronous turn. Both patterns can be repeated multiple times. [Videos available here: 10.6084/m9.figshare.30018262]

Interestingly, the male Indo-Pacific bottlenose dolphins (*Tursiops aduncus*) of Shark Bay, Western Australia, who have converged with humans in the formation of strategic intergroup alliances [17], also utilise synchrony in a cooperative context [18–20]. In this population, pairs and trios of allied males (first-order alliances) will perform displays of synchronised turns, leaps and surfacing bouts around single oestrus females that they are ‘consorting’ [18, 19, 21]. These displays often take the form of repeated turns behind and in front of the female, creating circular and variations of figure of eight patterns termed ‘butterfly’ displays (Fig. 1a). Additionally, males have been observed performing synchronous displays with no female present. In such instances, without the focal point of a ‘shared’ female, males tend to engage in successive parallel half turns termed ‘tango’ displays (Fig. 1b) or other types of displays, such as synchronised chin slaps on the water’s surface (ESM). Indeed, whilst many synchronous displays can loosely be categorised as butterfly or tango displays, males will often perform new movements that do not fit these categories, suggesting there is some degree of invention and spontaneity in these displays [19, 22].

First-order alliances are formed from within larger second-order alliances that comprise 4–14 similarly aged, generally unrelated males [23, 24]. Second-order alliances are considered the core unit of male social organisation and, once formed, can last decades [21, 25]. Whilst consorting, males can recruit their second-order allies to help defend and steal females from rival alliances [21]. Multiple second-order alliances can also develop weaker but still significant connections to establish third-order alliances, where males cooperate between groups to defend and capture females [17, 21, 26], thereby increasing the chance that males will have allies nearby. Across all alliance levels, bond strength is variable [26, 27], but fostering strong bonds with allies can provide strategic reproductive benefits [17, 28].

Allied males in this population use a variety of mechanisms to maintain and strengthen social bonds, such as physical affiliative contact (like rubbing and petting) and vocal exchanges [26, 29, 30], which are also commonly used as social bonding mechanisms in other species [31–36]. Past evidence from the Shark Bay dolphins suggests they might also use motor and acoustic synchrony as a tool to express social bonds [18–20, 22, 37]. However, only synchronous surfacings have been studied in detail [18, 19]. These occur most often between more strongly bonded first-order allies and may help maintain and strengthen social bonds, as well as signal alliance unity [18]. Fine-scale analysis of synchronous surfacings showed that dyads with weaker bonds synchronised with greater precision, suggesting that males who are less often together and not synchronous as often are more motivated to synchronise when they are together [19]. Males in this population also engage in acoustic synchrony in a cooperative context, with acoustic synchrony occurring at higher rates among more strongly bonded males [37]. Synchronous motor displays often (but not always) occur around a consorted female and, while they may play a role in social bonding, an alternative hypothesis is that males are signalling either their individual reproductive quality or alliance strength through displays. For instance, in the aforementioned fiddler crabs, females prefer males performing leading waves [38] or, in humans, observers perceive synchronous groups as more unified [13, 14].

Here, we test several hypotheses for synchronous displays in a cooperative context and explore additional factors that could affect display performance to help understand what proximate mechanisms might facilitate the occurrence of synchrony. Using a 40-year behavioural dataset of association history, combined with recent aerial video footage of synchronous displays, we investigate how male dolphin social relationships, social context and female presence affect the duration, intensity, and precision of synchronous displays. Affiliative behaviours, such as grooming in primates or petting in cetaceans, are known to increase the strength of social bonds [30, 39]. If males use synchronous displays to maintain social bonds, we predict a link with the presence of affiliative behaviour, such that synchrony will be more intense and last longer after affiliative behaviour has occurred. Further, if displays have the same nuance as surfacings in that they strengthen relationships between males with weaker social bonds, we expect weaker bonded males to execute synchrony with greater precision. Alternatively, if males use synchrony as a signal of reproductive fitness or coercion directed at a female, we expect the precision and intensity of synchrony to be greater when males are in the field of view of their consorted female.

## Methods

### Study system

Data for this study were collected from a population of Indo-Pacific bottlenose dolphins (*Tursiops aduncus*) in the eastern gulf of Shark Bay, Western Australia. Research on this dolphin population has been carried out on a near-annual and seasonal basis (usually austral winter and spring) since 1982 (Shark Bay Dolphin Research; www.sharkbaydolphins.org) [21]. The study was carried out in compliance with the ethics policies of the University of Bristol and the University of Western Australia. Permits for the scientific use of animals were obtained from the Department of Biodiversity, Conservation and Attractions (DBCA), Western Australia.

### Behavioural data collection

Behavioural synchrony data for this study were collected between 2018 and 2023. Video data included observations of 51 male dolphins from 11 different second-order alliances and 11 females, of which 30 males from 8 second-order alliances and 9 females were used in the final dataset (Table S1). In 2023, aerial video data were collected during focal follows of consorting groups (two to three allied males herding a single adult female) using a DJI Mavic 3 Classic Unoccupied Aerial Vehicle (UAV) (filming at 3840 × 2160-pixel resolution and 60 frames/second, ∼ 30 m altitude with a ∼ 90° camera angle). During these follows, the consorting group was followed for 1–2 h with the UAV (with brief breaks between UAV flights to change batteries), whilst synchronised boat-based voice notes (recorded on to a TASCAM DR-680 MKII multi-track recorder) were used to identify individuals and describe surface behaviour. Between 2018 and 2022, aerial video data were collected using a DJI Phantom 4 Pro + UAV (filming at 3840 × 2160-pixel resolution and 30 frames/second, at ∼ 30 m altitude and ∼ 90° camera angle) as part of focal follows lasting ∼ 60 min. These follows were structured similarly to those of 2023, with the UAV videoing a focal group and time-synchronised voice notes being recorded. However, focal follows from 2018 to 2022 were not exclusive to consorting groups.

### Quantifying motor synchrony

Previously, motor synchrony in the Shark Bay dolphins has only been studied via synchronous surfacings and defined as two, or sometimes three, individuals simultaneously breaking the surface [18, 19]. This has been quantified either by eye in the field or in the review of boat-based video recordings. For this study, we used a similar definition of two or more individuals initiating the same movement simultaneously, but we established a time threshold within which the movement needed to occur for it to be considered a synchronous event to minimise observer bias. Whilst two males performing a synchronous surfacing generally break the surface within 0.2 s of each other [18, 19], synchronous displays contain more complex, multi-dimensional movements, such as turns, leaps and dives. A preliminary set of video data containing motor synchrony displays was assessed by eye and the start (initiation of movement) of each male’s synchronous turns and surfacings were recorded. When visualising the distributions of time intervals between the synchronous movements of individuals from this preliminary dataset, most males began their synchronous movements within 0.4 s (x̄ ± SD = 0.23 ± 0.26, *n* = 11) of each other. We therefore set our synchronous threshold at 0.5 s: where two or more males had to perform the same behaviour *within* 0.5 s of each other for the behaviour to be classified as synchronous. This was further supported by a larger dataset of the time intervals between all the synchronous turns (x̄ ± SD = 0.22 ± 0.14, *n* = 161). This also aligns with other studies, for example, testing common bottlenose dolphins (*T. truncatus*) in a cooperative button pressing task, where pairs of dolphins synchronised the pressing of their buttons within 0.5 s of each other on average [40].

Whilst males primarily perform stereotypical butterfly and tango displays (Fig. 1), they also seem to incorporate novel inventions into their displays [18, 19, 21, 22]. This variation makes discrete categorisation of synchrony challenging. To resolve this, motor synchrony was quantified as individual elements within a display, and these individual elements were classified into one of four distinct types: a full turn (a sustained turn of the rostrum through more than 90°), a half turn (a sustained turn of the rostrum through less than 90°), a surfacing (break or dive through the water’s surface), and miscellaneous (an exaggerated movement of the body that does not result in a sustained change of orientation) (see Table S2a for more details).

### UAV video analysis

UAV video recordings of motor synchrony and time synchronised boat-based voice notes were rendered into one video (1280 × 720-pixel resolution) using DaVinci Resolve [41]. Using the event logging software BORIS [42], a total of 24 follows (∼ 15 h) were annotated for the occurrence of synchrony and other social behaviours. Synchronous elements were recorded as point events, where we marked the start of the synchronous movement and noted the ID of each participating male. For each synchronous full and half turn, this was the last frame that each male remained in his previous orientation (usually straight) before the turn was initiated. This enabled us to calculate the time lag between each male starting their turn and thus generate precision measurements. Precision measurements were taken from every turn where all participating animals were clearly visible and not overlapping. Precision measurements were only extracted from turns, as they typically occurred in the horizontal plane, providing unambiguous identification of when the turn started. When quantifying synchronous events for consorting groups, we also recorded if the males performed the synchronous element within the field of view of the female consort. Female field of view was defined as a 300° area around her rostrum, extending 4 m away (see Figure S1). This was based on the field of view of the closely related and morphologically similar common bottlenose dolphin [43], and visibility estimates were made from underwater observations in the field.

Social behaviours were recorded as state events, where we marked the start and end time of each type of social interaction. Social behaviours were categorised into three classes: affiliative, aggressive and sexual, with the latter category reserved exclusively for male-female interactions, despite the occurrence of male-male socio-sexual behaviours (see Table S2b for more details). The start time was recorded at the initiation of physical contact (or acceleration in aggressive chases) and the end time was recorded when physical contact ceased (or dolphins decelerated). For each social event, the participating individuals were recorded. In addition, if another group of animals joined the focal group, the composition of this group was recorded. All video data were annotated for social and synchronous behaviours by one observer (SHC) and then a second observer (DP) annotated approximately 20% of the data (5 follows/∼3 h) for the occurrence of synchronous events only. An inter-observer reliability analysis was then conducted using the intraclass correlation coefficient (ICC) function for two-way models within the *irr* package [44] in R [45], and we found excellent agreement for all measures of synchrony between the two observers (Table S3).

### Synchrony bout classification

To reflect the fact that synchrony occurs in displays, the discrete synchronous elements (half turns, full turns, surfacings, and miscellaneous) annotated in BORIS were grouped into bouts. To determine a bout threshold, time intervals between synchronous elements were extracted and a changepoint analysis was used to determine an inter-element interval threshold that would provide the cut-off point between synchronous elements within the same bout and the next bout. A changepoint analysis identifies changes in the trends of certain parameters (e.g. mean) within a dataset [46], and this can be used to categorise animal movements into discrete behavioural types or events [47, 48]. Based on the last 40 years of observation of the population, most male synchronous displays involve either repeated full turns around the female (e.g. a butterfly display) or repeated half turns (e.g. tango display). To reflect this, and preserve previous definitions of synchronous displays, a synchronous bout had to include at least two turns (half or full) for the bout to be used in further analysis. Therefore, we conducted a changepoint analysis on the time intervals between synchronous turns using the ‘CROPS’ penalty and ‘PELT’ method [49] within the *changepoint* package [46] in R 4.4.1 [45]. The threshold between bouts was determined to be 18 s, and a second changepoint analysis of all synchronous elements supported this threshold (Figure S2). Therefore, any synchronous elements performed (by the same group of males) within 18 s of each other was classified as being part of the same synchronous bout, where the bout contained at least two synchronous turns. Traditional tango displays would then be reflected in bouts with more half turns, and butterfly displays by those containing more full turns.

### Response measures of synchrony

For each synchrony bout, *bout duration* was calculated as the time in seconds (s) between the start of the first and last element within a bout. To represent the intensity of a synchronous bout, the number of elements within the bout were divided by its duration to give *element rate*. To measure *precision*, the time lag (s) between each male starting their turn was calculated between each dyad (i.e. if two males were engaging in synchrony there was one dyadic time lag; if three males were engaging in synchrony, there were three dyadic time lags). Finally, we also calculated the number of elements each male participated in per bout (for the consorting male trios only). We therefore established four dependent variables for synchrony in total. Two of these variables were calculated at the level of the synchronous group (2 to 3 participating individuals): *bout duration* and *element rate*. One measure was calculated between dyads (between each of the participating individuals): *precision*. Lastly, one measure was calculated at the level of the individual: *bout participation – the proportion of elements in which an individual participated per bout*.

### Social and demographic data

Long-term survey data was used to calculate social bond strength between males in this study. Survey data are collected between April and November each year, which includes the peak mating season (September to November), systematically throughout our current 600 km study area. A survey is a minimum 5-minute observation of dolphin group composition (as defined by the 10 m chain rule, where dolphins within 10 m proximity of any other are considered to be part of the same group [50]), during which predominant group behaviour is also recorded. Individual identification is conducted in the field by experienced researchers and then later confirmed using photographic identification of unique body features (e.g. dorsal fin shape, body markings and scars) [50]. Given the strong fission-fusion grouping patterns in bottlenose dolphin societies, association indices are a measure of bond strength and reflect true social preferences [51, 52]. Here, we use the Simple Ratio Index (SRI) [53] in the R package *asnipe* [54] to calculate social bond strength between individuals. SRI provides a dyadic measure of time spent together; with 1 for animals always seen together and 0 for animals never seen together. To calculate SRI, only group composition from the first 5 min of a survey was used to ensure association measures were comparable across surveys. The average number of surveys used per individual was 59 (range: 4–204). Resights (where the same group is encountered within 2 h) were excluded, as were surveys where foraging was the primary behaviour, as aggregation around food resources may not reflect true social preferences [17].

Males tend to be considered sexually mature at 15 years old or when they begin consorting [24]. For the males in this study, we calculated SRI from either the date the youngest male turned 15, or the date they were first observed consorting a female (whichever was earliest) up to the present date (i.e. up to the date that the motor synchrony data was collected). For trios of consorting males, we scored the male with weakest average SRI within the first-order as the odd-male-out (OMO).

### Statistical analysis

#### Factors affecting synchrony performance

To examine the factors affecting the performance of synchronous displays, we built linear mixed-effect models using the *lme4* package [55]. The three response variables were (a) *bout duration*, (b) *element rate* and (c) *precision*.

For the *bout duration* and *element rate* models, the continuous predictor variables were: average SRI of male participants, average age of male participants (in years), the number of male participants (2 or 3) and the number of other (non-participating) males that were present. We also included alliance level as a categorical predictor (first, second, third or unallied) and whether the participating males engaged in affiliative, aggressive or sexual behaviour in the 2 min prior to the synchrony bout – all as binary variables (0 = no, 1 = yes) (see behavioural ethogram in Table S2). We also included whether non-participating males who were present engaged in sexual behaviour in the 2 min prior to the synchrony bout as a binary variable (0 = no, 1 = yes). For the *precision model*, we included average SRI of the male dyad, average age of the male dyad, the number of male participants (2 or 3), the number of other (non-participating) males that were present and distance in body lengths between males when they turned as continuous predictors. As categorial predictors, we included alliance level (first, second, third or unallied) and the turn type (if the synchronous turn was half or full) and, as binary variables, we included whether the participating males engaged in affiliative, aggressive or sexual behaviour in the 2 min prior to the synchrony bout (0 = no, 1 = yes) and whether the males turned in the same direction (0 = no, 1 = yes).

For all *bout duration* and *element rate* models, to account for the non-independence of bouts within the same follow and repeated measures within the same group, follow ID and second-order alliance membership were included as random effects. In a few instances, participating males were from different second-order alliances. In these limited cases, if the participating males were first-order allies e.g. consorting a female, but were from different second-order alliances, then they were given a unique ID code e.g. X1. If the participating males were not a first-order alliance e.g. they were not consorting a female, then they were given the code XX. For the *precision* models, the ID of both males in the dyad are included as random effects, along with follow ID. The *bout duration* model showed significant deviation in the distribution of residuals and was therefore log-transformed. Similarly, the *element rate* model showed deviation in the distribution of residuals, so the response was log-transformed. All models were then standardised and ranked using Akaike Information Criterion (AIC) using the ‘dredge’ function from the *MuMIn* package [56]. For the *bout duration* and *element rate* models, model averaging was performed across top models with a ΔAIC of < 2 using the model.avg function within *MuMIn* [56]. For the *precision* models, linear mixed-effects models do not adequately address the non-independence between the male ID random factors. In contrast, multi-membership generalized linear models that incorporate a node dependence term are more effective in accounting for the non-independence [57]. Therefore, after standardising and ranking the *precision* model, we rebuilt our top model with ΔAIC of < 2 as a multi-membership generalized linear model with a node dependence term for each male ID, and follow ID as random effect using the MCMCglmm function in the *MCMCglmm* package [58]. However, the top model was slightly over the ΔAIC < 2 threshold (i.e. ΔAIC = 2.09), but it contained a predictor of interest, so we refitted a simplified *precision* model containing only the single predictor of interest and the random effects and compared it to the null model (intercept only model) via likelihood ratio tests (Table S4). As the predictor of interest significantly improved model fit, we rebuilt the model as a multi-membership generalized linear model using MCMCglmm to account for the non-independence of dyads.

#### Social bond strength and synchrony participation

To investigate Connor et al.’s [18] finding that, within first-order alliances, the odd-male-out (OMO) in association strength matched the OMO in synchrony participation, we built a model using the *glmmTMB* package [59]. The response variable was *bout participation* per male, and we weighted each observation with the total number of elements per bout. The predictor variable was OMO status (0 = male does not have weakest average SRI in his first-order, 1 = male had the weakest average SRI in his first-order). Male ID and bout ID nested within follow ID were included as random effects. We specified a zero-inflation component with an intercept-only model (*ziformula* = ∼ *1*), allowing for a constant probability of observing excess zeros across all observations.

#### Synchrony and subsequent social behaviour

To investigate whether synchronous displays affect the likelihood of males participating in affiliative behaviour, we built a binomial generalised linear mixed-effects model within the *lme4* package [55]. The response variable was whether affiliative behaviour started within the two minutes following the end of a bout of synchrony (0 = no, 1 = yes). Bout duration and element rate were included as fixed effects, and follow ID and second-order alliance membership were each included as random effects.

#### Effect of female presence on synchrony

To examine whether female presence affects male performance of synchrony, we used a reduced dataset (where only consorting males and a female were present) and built two linear mixed-effects models using the *lme4* package [55]. The response variables for each model were *bout duration* and *element rate*. We used a single continuous predictor of the proportion of bout elements within the female’s field of view. To account for the non-independence of bouts within the same follow, and repeated measures within the same group, follow ID and second-order alliance membership were included as random effects. The *bout duration* model showed significant outliers and deviation in the distribution of residuals; therefore, it was log-transformed. Similarly, the *element rate* model exhibited significant deviation in the distribution of residuals, so, it was also log-transformed.

We also built a multi-membership generalized linear model using MCMCglmm [58]. The response variable was precision, and we included whether males turned within the female’s field of view as a binary predictor variable (0 = no, 1 = yes). We included a node dependence term for each male ID, and follow ID as a random effect. To investigate whether the type of synchronous element (either a turn, as typically seen in displays or interpersonal synchrony, such as synchronous surfacings) influenced whether it was performed in the female’s field of view, we built a binomial generalised linear mixed-effect model within the *lme4* package [55]. The response variable was whether a synchronous element occurred in the female’s field of view (0 = no, 1 = yes) and the predictor variable was whether the synchronous element was a synchronous turn (half or full) or not (0 = no, 1 = yes). Bout ID was included as a random effect.

All statistical analyses were conducted in R 4.4.1 [45]. Model fit was assessed using the *DHARMa* package [60]. To test for collinearity, the variance inflation factor (VIF) was calculated for all independent variables using the *car* package [61]. All independent variables were retained as VIF values were less < 3 [62]. The raw data and model estimates were plotted with the packages *ggplot2* [63] and *effects* [61].

## Results

A total of 15 h 26 m and 7 s of Unoccupied Aerial Vehicle (UAV) video data across 24 follows (average of 38 m 35 s per follow) from 2018 to 2023 was analysed, yielding 968 synchronous elements (149 full turns, 128 half turns, 612 synchronous surfacings and 79 miscellaneous elements) from 30 different males across eight second-order alliances (see Tables S1&S5 for summary data). To account for the non-independence of synchronous elements within a display, any synchronous elements performed by the same group of males within 18 s of each other were classified as being part of the same synchronous bout (*see methods for further details*).

### Factors affecting synchrony performance

**Fig. 2:**
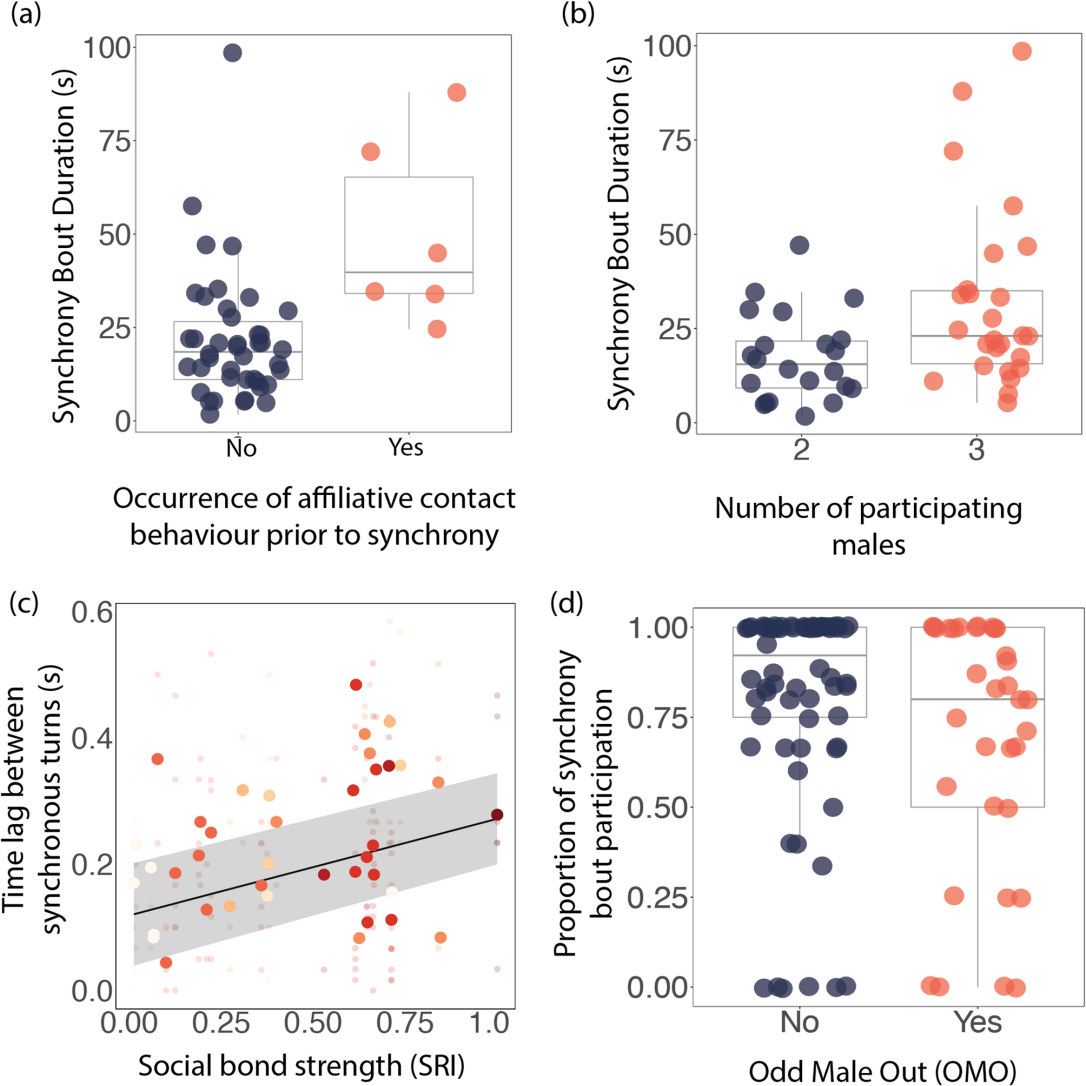
Social factors influencing synchrony. (**a**) Influence of affiliative contact behaviour (in the 2 min. prior to synchrony) on synchrony bout duration (seconds, (s)), and (**b**) Influence of the number of participating males on synchrony bout duration (s). Bout duration was calculated as the time difference between the first and last element in a bout. (**c**) Influence of dyadic social bond strength (calculated using the Simple Ratio Index (SRI)) on synchrony precision – measured as the time lag between a dyad’s synchronous turns. Each shade of red refers to a unique male dyad. (**d**) Difference in synchrony bout participation between the odd male out (OMO) and the remaining males in synchronising male trios. OMO was calculated as the male with the lowest average social bond strength within each trio. In (**a**), (**b**) and (**d**), raw data are shown as filled circles. In (**c**), small circles represent raw data and, for each dyad, their SRI and the average time lag between their synchronous behaviour is plotted as a larger circle. MCMC model estimates are indicated by the black line with 95% confidence intervals shown by the ribbon

We found that when participating males engaged in affiliative contact behaviour immediately prior (i.e. within 2 min.) to a bout of synchrony, the synchrony bouts were significantly longer in duration (Fig. 2a; Table 1). Additionally, trios of males performed synchrony bouts for significantly longer than pairs of males (Fig. 2b; Table 1). None of the other variables predicted synchrony bout duration. We did not detect effects of any of our variables on element rate within bouts (Table 1). Social bond strength had a significant positive effect on the time lag between a dyad’s turns (MCMC: posterior mean = 0.087, 95% CI [0.01, 0.16], pMCMC = 0.02): male dyads with weaker social bonds executed synchrony with greater precision than more strongly bonded dyads (Fig. 2c).

### Social bond strength and synchrony participation

Within every trio of synchronising males, we identified one male with the weakest average association index (SRI) as the odd-male-out (OMO). We found that whether a male was the OMO had a weak, non-significant effect on their participation in a synchrony bout (glmmTMB: β = -0.45, SE = 0.26, z = -1.7, *p* = 0.07; Fig. 2d).

### Synchrony and subsequent social behaviour

We found no effect of bout duration or element rate on the probability of males engaging in affiliative behaviour in the subsequent 2 min. after a synchronous event.

### Effect of female presence on synchrony

**Fig. 3:**
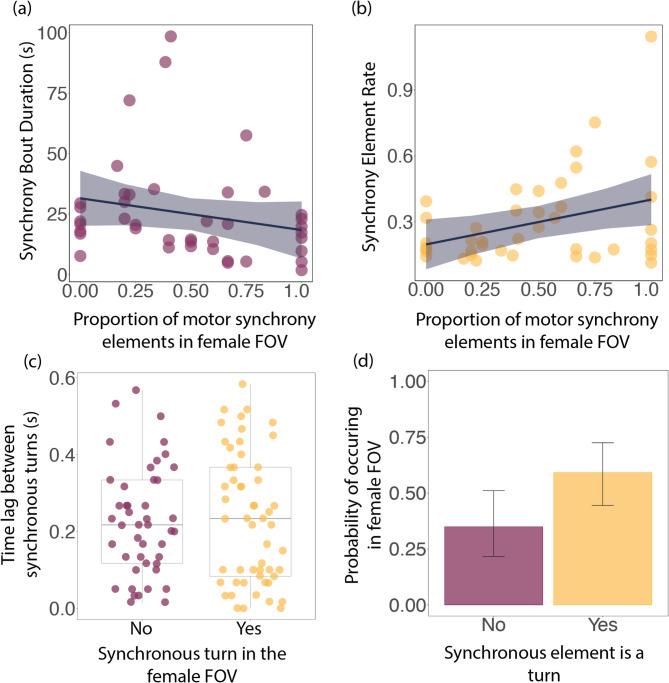
Male synchrony and female field of view (FOV). (**a**) Bout duration and (**b**) Element rate as a function of the proportion of motor synchrony elements in the female FOV. (**c**) Whether a synchronous turn occurred in the female FOV and how precise that turn was – measured as the time lag between a dyad’s synchronous turn. (**d**) Predicted probability of synchronous elements occurring in the female’s FOV. Synchronous turns include both full and half turns, and non-turns includes all miscellaneous and surfacing synchrony. In (**a**–**c**), the raw data are shown as filled circles. In (**d**), 95% confidence intervals are shown by error bars

When males performed a greater proportion of the synchronous elements of a display outside of the female’s field of view, bout duration was significantly longer (lmer: β ± SE = -0.800 ± 0.348, t(38.60) = -2.301, *p* = 0.027); Fig. 3a). We found that the proportion of bout elements within the female’s field of view had a positive, but non-significant effect on the element rate of synchrony (lmer: β ± SE = 0.458 ± 0.228, t(36.58) = 2.011, *p* = 0.052; Fig. 3b). There was no significant relationship between the synchronous turns that occurred in the female field of view and the precision of those turns (Fig. 3c). However, synchronous turns (half and full) did occur significantly more often within the female field of view than did interpersonal (surfacing and miscellaneous) synchronous elements (GLMM: β ± SE = 0.995 ± 0.342, z = 2.91, *p* = 0.0037; Fig. 3d).

**Table 1:** Results from mixed-effect models examining the effect of social factors on bout duration and element rate of synchrony

Response	Predictor	Effect	S.E.	CI (2.5%)	CI (97.5%)
*Bout Duration*	**Affiliation Before**	**0.927**	**0.356**	**0.229**	**1.625**
	**Number of Participants**	**0.571**	**0.241**	**0.098**	**1.044**
	Social Bond Strength	0.254	0.273	-0.281	0.789
	Aggression Before	0.207	0.388	-0.552	0.967
	Non-Participating Male Sexual Behaviour Before	-0.033	0.210	-0.445	0.380
	Number of Other Males	0.045	0.141	-0.232	0.322
*Element Rate*	Number of Participants	-0.239	0.207	-0.644	0.166
	Affiliation Before	-0.171	0.247	-0.655	0.312
	Participating Male Sexual Behaviour Before	0.032	0.110	-0.185	0.248

Parameter estimates were averaged over the top model set based on AIC selection (ΔAIC of < 2). Model averaging was performed across 8 (*bout duration*) and 5 (*element rate*) top models, respectively. S.E = adjusted standard error, CI = 95% confidence intervals. Bold values indicate significant results (i.e., confidence intervals that do not intersect zero)

## Discussion

We used aerial video footage to quantify synchronous displays among allied male dolphins and examined the social factors influencing their duration, rate, precision and participation. We found that allies engaged in synchronous behaviours for significantly longer following affiliative contact. Previous research based on synchronous surfacings suggested that motor synchrony has a social bonding function [18, 19]. Here we show that, for coordinated male displays, motor synchrony is exaggerated in affiliative contexts. This result mirrors a recent finding in chimpanzees, where play behaviour that strengthens social bonds (similar to affiliative contact in dolphins [29]) preceded coordinated action [64]. We also found that the precision of synchronous turns was predicted by a dyad's social bond strength; male dolphins with weaker social bonds engaged in significantly more precise synchrony. This complements previous findings in synchronous surfacings, that males with weaker bonds are motivated to engage in social bonding in the form of synchrony [19]. Further, bouts of synchrony were longer when they involved three males rather than two, a possible indication that coordination takes longer for three males.

When consorting a female, allies must work together continually, maintaining their bonds and guarding a female for extended periods (n.b. the longest documented consortship is currently ∼ 70 days, unpub. data). Affiliative contact may be the most effective mechanism to achieve bond maintenance but it requires males to be in proximity and swimming slowly, which is likely impractical when attempting to reduce the chances of female theft or escape. In contrast, synchronous displays can be performed at speed, with males remaining in their herding formation behind the female or switching positions with each synchronous turn. Motor synchrony therefore provides a mechanism for first-order allies to efficiently maintain their bonds whilst concurrently herding an oestrus female. Second- and third-order allies are also observed performing synchronous displays, but not in the context of consorting a female together. Yet, these displays tend to occur whilst males cooperate to defend each other’s female or in the context of joint aggression against other males (although we have also documented third-order allies engaging in synchronous displays with no female present and no joint aggression). The sole example of non-allied males engaging in a synchronous display was performed after they engaged in joint aggression towards another male. Therefore, at all alliance levels, males may use synchrony to ‘bond-on-the-go’, maintaining their social bonds whilst actively engaging in cooperation.

In this study, male dyads with the weakest social bonds performed synchronous turns with the greatest precision, a result that mirrors previous findings from synchronous surfacings [19]. Where an animal is capable of entraining to a stimulus, it is thought that the individual’s ability to execute this is driven by their attention and motivation [65]. Our findings suggest that males have more motivation to engage in synchronous displays and are paying more attention to their partner when they share a weaker bond. This is similar to findings in humans, where synchrony during conversations predicts motivation to deepen a relationship in strangers, but not in friends [66]. Given that males need to work together to gain access to females, their bonds with other males play a key role in their lifetime reproductive success [28]. It therefore follows that, when males share weaker bonds, they may pay more attention to one another’s movements and are more motivated to strengthen that bond compared to males with strong, well-established bonds. In contrast, we found that within a male trio, the OMO in bond strength participated in fewer elements per bout of synchrony. The same relationship was found in the original study of synchronous surfacing bouts [18]. Whilst not significant (perhaps due to the smaller sample size in our study), this trend highlights the possibility that strongly bonded males may engage in synchrony more often, despite weaker bonded males being more attentive and motivated during synchrony. In humans, the relationship between synchrony and affiliation is underpinned by oxytocin, the social bonding hormone [67–69]. Whilst such a relationship is hard to identify in cetaceans, experiments have shown oxytocin increases prosociality and trust in other animals [70, 71], and that affiliative contact between partners leads to oxytocin production [72–75]. Therefore, synchronous displays may reduce tension and facilitate cooperation during the competitive context of consortships.

Synchrony has a bonding function in this species, but could also serve as a signal of alliance unity or individual reproductive quality [18]. Yet, the receiver of this signal is not always clear; in approximately one third of synchrony bouts, males performed displays in the absence of a female consort. Notably, we found that synchronous bouts were significantly shorter when a female could observe more of the elements, which could imply that males terminate displays early once they have performed enough synchronous elements in front of the female. The rate at which males performed synchrony was not significantly impacted by whether females could observe more elements, perhaps suggesting that the intensity of synchrony doesn’t depend on whether females can observe it or not. Similarly, we found no evidence that males perform synchrony with greater precision in the female’s field of view. Allied male dolphins will also synchronise the production of their ‘pop’ vocalisations [20]. Pops are low-frequency pulses used by males to induce the female to remain close during consortships [76–78]. First-order allies with strong social bonds engage in higher rates of pop synchrony [37]; similar to our non-significant finding here, that the OMO participated in less synchrony. As males with strong social bonds experience the greatest reproductive success [28], vocal synchrony may also be used to facilitate cooperation and build strong bonds. Playback experiments have shown no differences in male and female responses to synchronous vs. asynchronous pops, supporting the hypothesis that the function of pop synchrony is likely intrinsic to the signallers, and that vocal synchrony evolved to promote social bonding among the allied males producing the synchronous sounds [77]. Assuming vocal and motor synchrony share a common function in these dolphins, the pop synchrony experiment would support our findings that motor synchrony serves a social bonding function. This would be in line with results in other delphinids where synchronous swimming has an affiliative function [79–81]. While possible that synchronous displays would have evolved to impress females, potentially signalling alliance unity or individual reproductive quality, they now appear to play a key role in male-male bonding.

An interesting finding was that trios of males performed longer bouts of synchronous displays compared to pairs while not adding more elements to these bouts. This may indicate that it is more challenging to coordinate synchronous displays among three versus two individuals. Although larger groups are theorized to be more intelligent [82–85], reaching consensus takes longer, as coordination becomes more challenging with more individuals involved [86–89]. Such theory and evidence from the broader field of collective behaviour often come from studies on groups that navigate their environment while maintaining cohesion [90]. Here, despite dolphins performing the complex task of synchrony during displays, we still see a similar link between group size and measures of coordination. It is also worth noting that the addition of just one individual makes a difference in synchronous displays, while in general group movement studies, we typically neglect such differences and look into larger variations in group size [90]. Local interactions and simple rules can lead to the emergence of complex collective outcomes in moving animal groups [91, 92]. Tasks and behaviours that appear complicated at first glance often consist of a sequence of elements that can be socially learned and may differ across cultures [93]. Therefore, the mechanisms that lead to the emergence of synchronous displays in the male dolphins of Shark Bay—and whether they rely on local phenomena, as well as whether the building blocks of these displays culturally differ across populations or even alliances—remain unknown. Such investigations would open new windows into the understanding of the coordination mechanisms of complex collective movements.

To summarise, we explored the social factors that affect the performance of synchronous displays that occur within the cooperative context of consortships or joint action between allied males. We found synchrony lasted longer after affiliative behaviour was observed, and was more precise among more weakly bonded allies, supporting the hypothesis that synchrony helps build and maintain social bonds. This supports recent experimental evidence that acoustic synchrony also maintains social bonds in this population [77]. Leading hypotheses propose that the social use of rhythm evolved in humans to promote cooperation and social bonding [94–97], to advertise coalition quality [94, 98], and to attract mates [94, 96, 98]. Evidence on cooperative synchrony in fireflies supports the mate attraction hypothesis [1, 4, 5]. Our work provides complementary evidence that motor and vocal synchronous displays in dolphins have evolved to facilitate cooperative social interactions and maintain social bonds, without excluding the possibility that they play a role in advertising coalition quality to the female.

## Supplementary Information

Below is the link to the electronic supplementary material.

Supplementary Material 1

## Data Availability

All data generated or analysed during this study are included in this published article and its supplementary information files. Datasets and R code reported in this paper and videos of synchronous displays are available here: 10.6084/m9.figshare.30018262.
