# Supplementary material for: Allied male dolphins use synchronous displays to strengthen social bonds in a cooperative context
Source: Mov Ecol. 2025 Nov 21;13:84. doi: 10.1186/s40462-025-00603-z (PMC12636197; doi:10.1186/s40462-025-00603-z)
Supplement: Supplementary file 1 — Supplementary Material 1 [file 40462_2025_603_MOESM1_ESM.docx]

Supplemental information for **“Allied male dolphins use synchronous displays to strengthen social bonds in a cooperative context”**

**Sam Hill-Cousins, Emma Chereskin, Simon J. Allen, Richard C. Connor, Michael Krützen, Danai Papageorgiou and Stephanie L. King**

*Correspondence: Sam Hill-Cousins [sam.hill-cousins@bristol.ac.uk](mailto:sam.hill-cousins@bristol.ac.uk) and Stephanie L. King, [stephanie.king@bristol.ac.uk](mailto:stephanie.king@bristol.ac.uk)

**Videos of synchronous displays are available here: 10.6084/m9.figshare.30018262.**

**Table S1. Summary of aerial video data collected for the 11 second-order alliances in this study.** Each second-order alliance is represented by a two-letter code. The number of males per second-order alliance is provided, along with the total duration (hours:minutes:seconds) of footage for each alliance and the years during which they were filmed.

| Second-Order Alliance | # of Males in video data | Duration Of Footage | # of males in final dataset | Study years observed |
| --- | --- | --- | --- | --- |
| RR | 3 | 05:36:07 | 3 | 2021, 2023 |
| SB | 6 | 01:48:11 | 5 | 2021, 2023 |
| LL | 4 | 01:14:17 | 4 | 2021, 2023 |
| EC | 5 | 01:32:42 | 4 | 2023 |
| KS | 5 | 04:31:47 | 3 | 2019, 2021, 2023 |
| SC | 3 | 00:50:51 | 0 | 2023 |
| HG | 6 | 02:25:34 | 5 | 2021, 2022, 2023 |
| XF | 2 | 00:19:58 | 2 | 2021 |
| PB | 3 | 00:13:46 | 3 | 2018 |
| AC | 4 | 02:01:52 | 1 | 2023 |
| PD | 3 | 01:09:46 | 0 | 2023 |
| BB | 5 | 00:45:23 | 0 | 2021, 2023 |
| Unassigned | 4 | 01:00:03 | 4 | 2021, 2023 |

**Table S2. Synchronous and social behaviour ethogram.** (A) Synchronous behaviours were recorded as one of four discrete categories: half turn, full turn, surfacing or miscellaneous. (B) Social behaviours were recorded as one of three categories: affiliative, aggressive or sexual.

| **Behaviour** | **Example** | **Description** | **Measurement** |
| --- | --- | --- | --- |
| 1. **Synchrony** | |  | **Occurrence** |
| **Half Turn** | 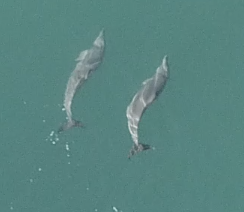 | Execute a sustained turn of their rostrum through < 90°. Here, two males perform a half turn. | At the first sustained change in orientation. |
| **Full Turn** | 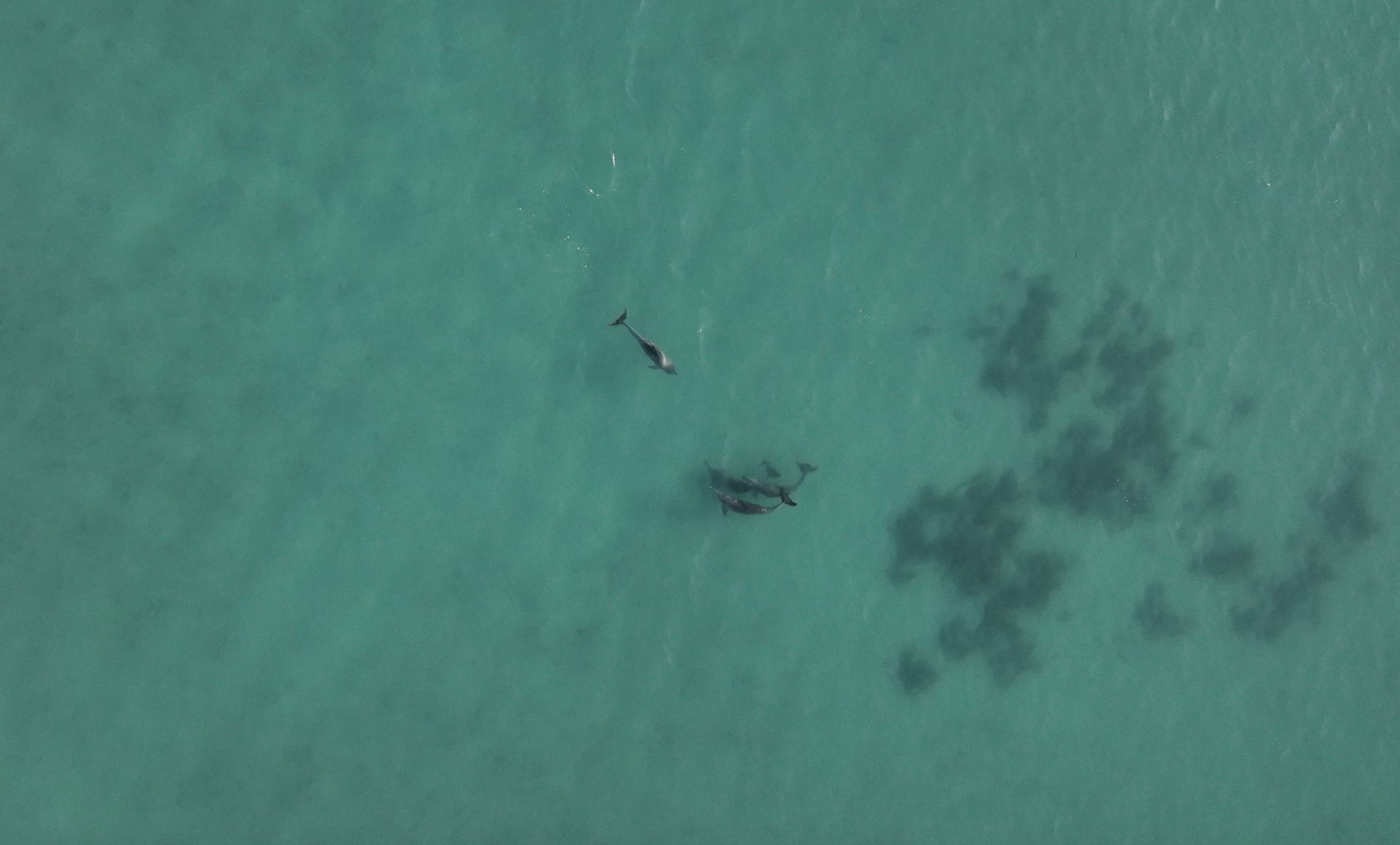 | Execute a sustained turn of their rostrum through >90°. Here, three males perform a full turn. | At the first sustained change in orientation. |
| **Surfacing** | 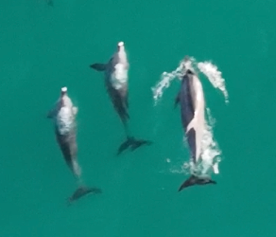 | Break or dive through the surface. Here, two males (left) perform a synchronous surfacing, whilst one male (right) misses the synchrony threshold (i.e. 0.5 s) | At the first break of water surface by animal’s dorsum. |
| **Miscellaneous** | 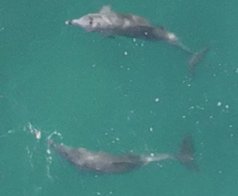 | Perform an exaggerated movement of the body that does not result in a sustained change of orientation. This category aims to encapsulate some elements of the invention in synchronous displays. Here, two males perform a synchronous body roll but do not change orientation. Other such behaviours can include ‘rooster struts’ - where males arch their head out of the water and rhythmically smack them into the surface of the water, or tail whacks – where dolphins repeatedly lift and smash their tail into the water. | At the start of movement. |
| 1. **Social** |  |  | **Start/End Point** |
| **Affiliative** | 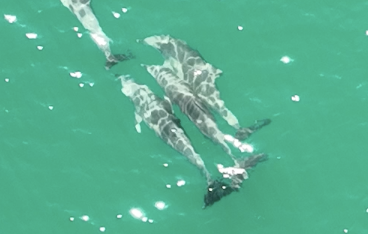 | A dolphin either pets (using their pectoral fin) or rubs one part of their body (e.g. keel) against part of another dolphin’s body (e.g. ventral side). Here, three males are petting. | At the point of first physical contact/ cessation of physical contact |
| **Aggressive** | 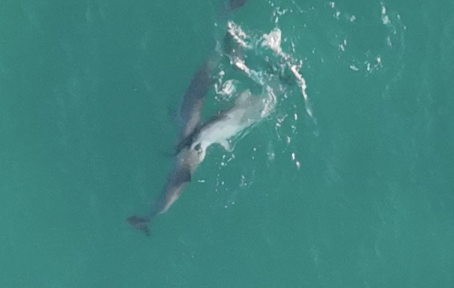 | An antagonistic interaction between two or more dolphins that can take the form of biting, charging, hitting, or chasing. Here, one male bites another. | At the point of first physical contact, or in the case of a chase, at start of acceleration/ cessation of physical contact, or deceleration. |
| **Sexual** | 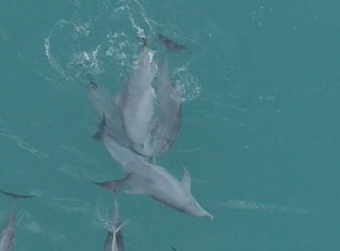 | Any prolonged physical contact between a male and female dolphin that can take the form of one of the above affiliative and aggressive interactions but typically directed at an animal’s genital slits. Here, three males probe the genitals of a female with their rostra. | At the point of first physical contact, or in the case of a chase, at start of acceleration/ cessation of physical contact, or deceleration. |

**Table S3. Summary of results of intraclass correlation coefficient (ICC) results for agreement between two independent observers’ measurements of synchrony.** Each measure of synchrony is presented (e.g. bout duration, element rate, precision), along with the Intraclass Correlation Coefficient (ICC), the lower (2.5%) and upper (97.5%) confidence intervals (CI) and the p value, Pr(ICC) of synchrony.

| **Measure of Synchrony** | **ICC** | **CI (2.5%)** | **CI (97.5%)** | **Pr(ICC)** |
| --- | --- | --- | --- | --- |
| **Bout duration** | 0.971 | 0.914 | 0.99 | <0.001 |
| **Element Rate** | 0.915 | 0.76 | 0.972 | <0.001 |
| **Precision** | 0.938 | 0.906 | 0.959 | <0.001 |

**Figure S1. Field of view of a female dolphin.** The blue area represents the visual field of an adult Indo-Pacific bottlenose dolphin (*Tursiops aduncus*), based on the ~300° horizontal field of view of their congener *T. truncatus* (Gunnars and Bruck, 2020). This field of view is expanded by length *2l*, where *l* represents the body length of an adult female dolphin (~2 m) in this population. This assumption is based on field observations estimating water visibility at 4 m.

**Figure S2. Changepoints for all synchronous elements time intervals (left) and all synchronous turn time intervals only (right).** Each changepoint is represented by a horizontal red line, with the end of this line matching the data point and time interval where a change in the trend of the mean of the data was identified. The dotted blue lines highlight the changepoint that converges across all three data sets, and which, after examination of resulting bouts, was used as the inter-element interval (IEI) that discerns elements from being in the same bout versus separate bouts. All changepoints (red lines) were (left) all synchronous elements: 6.657, 15.949, 30.964, 60.327, 101.251, 158.142 and (right) synchronous turns only: 17.801, 52.752, 112.679. Y-axis were limited to time interval = 200 s.

**Table S4. Results of likelihood ratios tests comparing top and models explanation of variance in precision.** Chi-squared tests were conducted on the deviance between the top model from model ranking and a null model. See methods for definition of term codes.

|  | **Model** | **Deviance** | **p-value** |
| --- | --- | --- | --- |
| i) | All Males |  |  |
| Full | lmer(Precision ~ SRI + (1 \| Male1) + (1\| Male2) + (1\| Follow) | -177.98 | 0. 02028 |
| Null | lmer(Precision ~ 1 + (1 \| Male1) + (1\| Male2) + (1\| Follow) | -172.59 |  |

**Table S5. Summary of synchrony data.** Sample size and summary statistics are presented for the full dataset and for the only consorting males reduced dataset. Bout duration was calculated as the time difference in seconds between the first and last synchronous element in a bout. Element rate was calculated as the number of elements in a bout divided by the duration. Precision measures were calculated as the time lag between a dyad’s synchronous turns.

| **Bout Type** | **# of Second- order alliances** | **# of Males** | **Total Bouts** | **Average Elements per Bout** | **Average Bout Duration (s)** | **Average Element Rate** | **# Precision Turns** | **# Precision measures** |
| --- | --- | --- | --- | --- | --- | --- | --- | --- |
| All males | 8 | 30 | 48 | 5.69 | 25.09 | 0.307 | 87 | 161 |
| Consorting males | 8 | 28 | 41 | 5.34 | 25.12 | 0.298 | 63 | 99 |

Note: For the precision models, there was one additional second-order alliance (XF, two males) that was not part of the bout duration or element rate models as the bout involved four males (two from the XF alliance and two unallied males) displaying together but not consistently as a group. Therefore, this did not allow for discrete bouts to be attributed to specific participants and left them unsuitable for duration and rate analysis. However, precision measures were still extracted from the synchronous turns to contribute to the precision model.
